# Searching for perceptual similarity within, and between, the (chemical) senses

**DOI:** 10.1177/20416695221124154

**Published:** 2022-09-22

**Authors:** Charles Spence

**Affiliations:** Oxford University, UK

**Keywords:** perceptual similarity, affective similarity, crossmodal congruency, chemical senses, crossmodal correspondences

## Abstract

In this narrative historical review, I want to take a closer look at the concept of perceptual similarity both as it applies within, and between, the chemical senses (specifically taste and smell). The discussion is linked to issues of affective similarity and connotative meaning. The relation between intramodal and crossmodal judgments of perceptual similarity, and the putatively special status of those odorants that happen to take on taste qualities will also be discussed. An important distinction is drawn between the interrelated, though sometimes distinct, notions of perceptual similarity and crossmodal congruency, specifically as they relate to the comparison of chemosensory stimuli. Such phenomena are often referred to as crossmodal correspondences, or by others (incorrectly in my view), as a kind of ubiquitous synesthesia.

## Introduction

At the start of his now-classic article from almost half a century ago,^1^ Amos Tversky (1977, p. 327) wrote that: “Similarity plays a fundamental role in theories of knowledge and behavior. It serves as an organizing principle by which individuals explain and classify objects, form concepts, and make generalizations. Indeed, the concept of similarity is ubiquitous in psychological theory. It underlies the accounts of stimulus and response generalization in learning, it is employed to explain errors in memory and pattern recognition, and it is central to the analysis of connotative meaning.” While Tversky likely had the similarity between visual, and perhaps also between auditory, stimuli in mind (see also Mehrabi et al., 2019, on auditory perceptual similarity), those scientists interested in the topic of multisensory flavor perception (such as, e.g, your author) are presumably entitled to ask whether the same claim also applies when it comes to thinking about similarity in (and between) the chemical senses (here focusing, in particular, on olfactory and gustatory stimuli). This is the question that I will address in this narrative historical review. The topic takes on added contemporary relevance given the growing interest in flavor pairing that has been seen in recent years (e.g., see Coucquyt et al., 2020; Spence, 2020b, 2022b, for reviews).

### Assessing the Similarity of Culinary Spices and Other Seasonings

**Figure 1.**
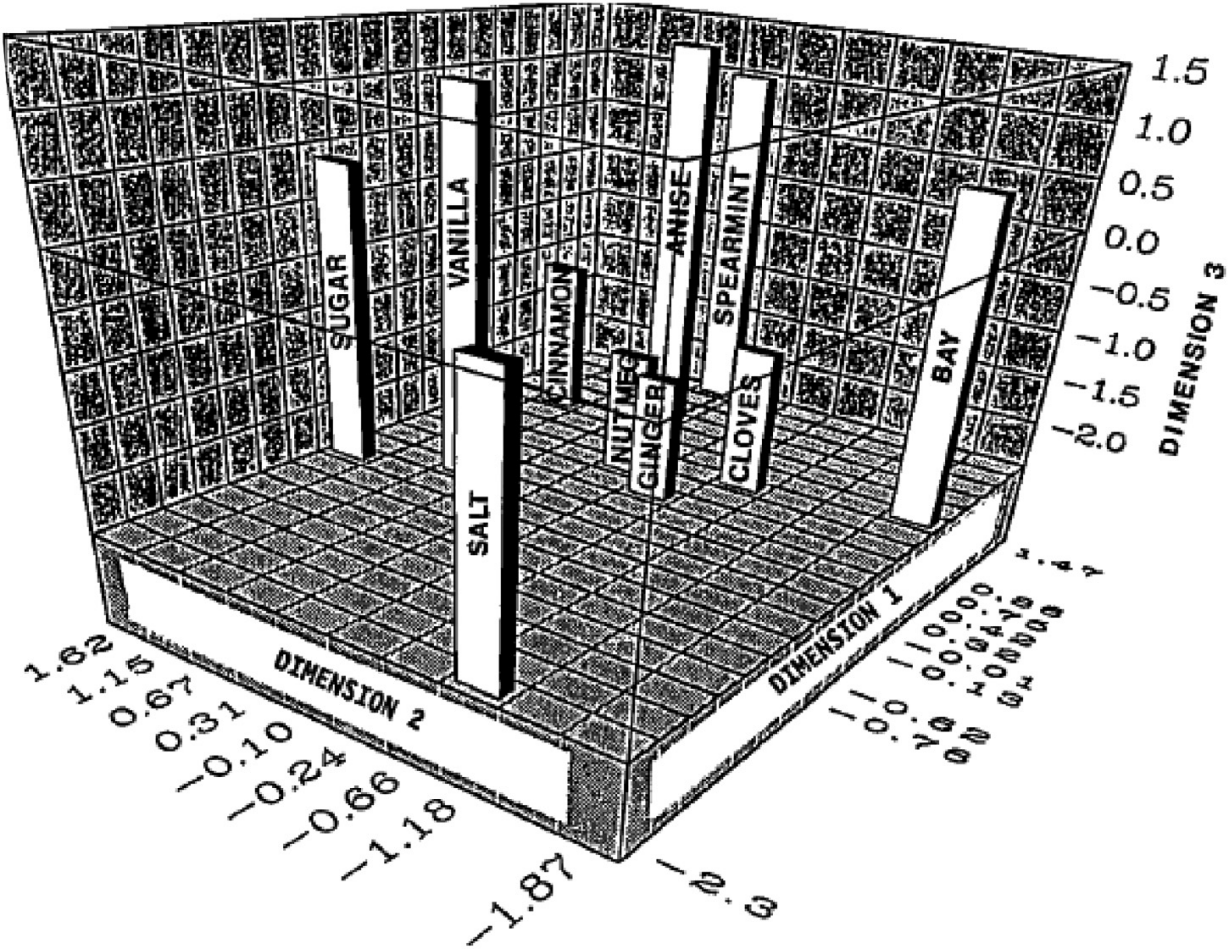
Multidimensional scaling (MDS) configuration of spices achieved by ALSCAL for all 70 participants. Figure reprinted from Blank and Mattes (1990). [Granularity present in original journal image.]

In one of the first empirical studies designed to address the question of similarity within the chemical senses, Blank and Mattes (1990) had two groups of participants (one group of White North American participants and the other group of non-White participants who mostly came from outside the United States; *N*  =  70 in total) rate the pairwise similarity of 45 pairings of 10 spices.^2^ The stimuli included salt and sugar granules, vanilla and anise extract, dried bay leaf, dried crushed spearmint leaves, ground nutmeg, cloves, cinnamon, and ginger. The participants were given no specific guidance as to the basis on which they were supposed to rate the similarity of the stimuli that they first smelled and then tasted. The participants responded using a nine-point scale anchored with the terms “very similar” and “very different” (see Figure 1 for the results).

According to Blank and Mattes (1990), a majority (69%) of the variance in the data that was collected could be explained by just three dimensions. Discussion with the study participants led to the suggestion that one dimension (identified as Dimension 1 in the figure) was best understood as coding stimulus intensity, Dimension 2, it was suggested, coded the compatibility of the spice with sweetness, while Dimension 3 was associated with the degree of the bitter taste of the stimulus. One question that immediately crops up here is why the participants did not rate salt and sugar as being similar, given that both stimuli are typically encountered as crystalline white substances (note that they were, in fact, presented in granular form) that lack any discernible odor. Perhaps, though, the fact that the participants were instructed to smell and then taste the stimuli was sufficient to direct their attention to the taste/flavor (rather than focusing on the visual appearance of the stimuli).

While the results of Blank and Mattes’ (1990) study would appear to support the claim that people can make meaningful judgments of the similarity of pairs of chemosensory stimuli, the basis on which they made their similarity judgments remains unclear. *A priori*, one could imagine that the participants would have based their decisions on perceptual similarity, on affective similarity, on similar patterns of usage of the “spices” in recipes, on similar visual appearance cues (as was just mentioned), or perhaps even on the extent to which the stimuli happen to engage the olfactory versus gustatory systems.^3^

Meanwhile, in an earlier study, Jones et al. (1978) had their participants (*N*  =  22 students) assess the similarity/confusability of the 55 possible combinations of 11 common herbs or seasonings (comprising basil, bay*,* celery*,* marjoram*,* mint, oregano*,* parsley*,* rosemary*,* sage*,* tarragon*,* and thyme) on a nine-point similarity scale. In this case, the data supported a unidimensional solution for similarity judgments plotted against relative confusion in recognition memory. That said, one might want to consider the extent to which the context in which the stimuli are presented affects the similarity space that is obtained (cf. Cleland et al., 2002; Yearsley et al., 2021). Elsewhere, research from Dalton et al. (2008) revealed that three dimensions (corresponding to the evaluation, potency, and activity dimensions of semantic differential theory) were needed to account for the majority (53%) of the variance in semantic differential-type judgments of a range of 30 distinct olfactory stimuli.

## Distinguishing Congruency From Similarity in the Case of the Chemical Senses

Spence (2022a) raised the question of whether we should think of those olfactory stimuli that take on taste properties (and which could potentially be used as sweet replacers; Blank & Mattes, 1990; Fial, 1978) as an example of crossmodal/semantic congruency (Amsellem & Ohla, 2016), crossmodal correspondence, and/or perhaps perceptual similarity. This is more than merely a matter of semantics: For understanding what exactly we are talking about is important given the suggestion that it is the perceptual similarity between combinations of olfactory and gustatory stimuli that determines the magnitude of any oral referral (Schifferstein & Verlegh, 1996). In turn, the extent to which the odorant is referred to the oral cavity likely predicts the extent of any odor-induced taste enhancement (OITE; Schifferstein & Verlegh, 1996; see Spence, 2016, for a review of the phenomenon of oral referral).

In an intriguing early study, Schifferstein and Verlegh (1996) attempted to distinguish between the concepts of congruency and similarity in the context of the chemical senses. The aroma of vanilla and the taste of sugar, for example, are both congruent and also perceptually similar (i.e., many people describe both as “sweet”; see Blank & Mattes, 1990; Spence, submitted). By contrast, while acetic and citric acid are perceptually similar (in that both are acidic) they are not congruent (in the sense that they do not typically co-occur together in foods; see Table 1).^4^ At one point, Schifferstein and Verlegh (1996, p. 87) write that: “Therefore, congruency or pleasantness ratings cannot replace similarity ratings in accounting for odor-induced taste enhancement.” They also highlight the different underlying mechanisms between congruency and similarity (with the former, in their view at least, being culturally determined)^5^ and place a special importance on the perceptual similarity of combinations of olfactory and gustatory stimuli (see also Frank et al., 1991, for similar conclusions; and Murphy & Cain, 1980, for discussion of the “harmony” of certain odor-taste mixtures).

**Table 1.** Putative combinations of olfactory and/or gustatory stimuli that vary in terms of their perceptual similarity and congruency—the latter defined by Schifferstein and Verlegh (1996) as stimulus pairings that co-occur in flavorful stimuli.

	Perceptual similarity	
	Yes	No
Congruent	Vanilla odour + sugar	Sweet + sour
Incongruent	Acetic acid + citric acid	Chicken broth aroma + sweetness

### Does Perceptual Similarity Exist Across the Senses

At this point, it is important to note that not everyone even believes that perceptual similarity judgments between the senses are necessarily possible. In particular, the early German psychophysicist, Hermann Ludwig Ferdinand von Helmholtz (1821–1894) once wrote that: “the distinctions among sensations which belong to different modalities, such as the differences among blue, warm, sweet, and high-pitched, are so fundamental as to exclude any possible transition from one modality to another and any relationship of greater or less similarity. For example, one cannot ask whether sweet is more like red or more like blue … Comparisons are possible only within each modality; we can cross over from blue through violet and carmine to scarlet, for example, and we can say that yellow is more like orange than like blue!” (Helmholtz, 1878/1971, p. 77). Given that Helmholtz (1878/1971) makes no explicit mention of the chemical senses, it is difficult, in hindsight at least, to know whether he had the same opinion regarding comparisons amongst the chemical senses (e.g., that judging the similarity of olfactory and gustatory stimuli is equally futile). Furthermore, given that one can talk about both perceptual and affective similarity (see Spence & Di Stefano, 2022), it would seem reasonable to assume that Helmholtz was referring to the question of perceptual similarity in the above-mentioned quote. Here, though, one might want to consider whether Helmholtz intended his claim to refer only to the proper sensible rather than to what Aristotle (1907) referred to as the common sensible (see Everson, 1995; Owens, 1982; Spence & Di Stefano, submitted).

That said, not everyone necessarily agrees with Helmholtz. So, for example, a seemingly contradictory position was forwarded by Lawrence Marks (2011, p. 52) when writing about: “perceptual similarities between and among sensory experiences in different modalities. Much as the color aqua is more similar phenomenologically to cerulean than to pink, the flavour of lime more similar to lemon than to banana, so too are low notes played on a bassoon or an organ more like dark colors such as brown or black than bright colors such as yellow or white, while the higher notes played on clavier or a flute resemble yellow or white more than brown or black.”

Evidence that might, at first, appear to contradict Helmholtz’s (1878/1971) assertion comes from those studies in which the participants were instructed to pick the color (typically from a restricted range of options) that is most strongly associated with each one of the basic tastes, or vice versa. The majority of people will, for example, tend to agree that a red drink, or a red or pink color patch, looks (or is associated with) sweet rather than, say, sour (see Spence et al., 2015, for a review of the literature on color-taste correspondences; Huisman et al., 2016; O’Mahony, 1983; Woods & Spence, 2016). It is, though, important to note that while consensual responses such as these are often obtained under such forced choice experimental conditions, this does not necessarily mean that the participants in the studies concerned thought that the stimuli that they paired together were perceptually similar to one another (see Spence & Levitan, 2022, on this point). For example, people’s responses might instead merely reflect an expected, or predictive, relationship (i.e., associative learning), such as, for example, that if I see a red drink then I expect it to taste sweet (cf. Baeyens et al., 1990). Multisensory source objects might also underpin a number of such crossmodal correspondences between color (e.g., red) and a specific smell (e.g., strawberry aroma; see also Spence, 2020c). This, one might wish to think of as the semantic account.

The associative learning of such color-taste mappings has been documented in infants of no more than a few months of age (Reardon & Bushnell, 1988; cf. Fernandez & Bahrick, 1994). It is, however, important to note that such associative learning does not necessitate that the component stimuli, the color “red” and the taste of sweetness, are perceived as being perceptually similar (nor that they start to become more perceptually similar), merely that they tend to co-occur in the environment (see Foroni et al., 2016), and that we internalize such environmental statistics (Barlow, 2001). To illustrate the point, consider only how people might well want to pair a barking sound with the picture of a dog (rather than a cat; Wegner-Clemens et al., 2022) without necessarily wanting to assert that the auditory and visual stimuli are themselves perceptually similar.

## Crossmodal Correspondences Capture Multiple Kinds of Crossmodal Relationship

Crossmodal correspondences have been defined as a tendency for a feature, or attribute, in one sensory modality, either physically present, or else merely imagined, to be matched (or associated) with a sensory feature in another sensory modality (see Spence, 2011, for a review). More than two decades ago, Martino and Marks (2001, p. 61) suggested that: “Weak synaesthesia is characterized by cross-sensory correspondences expressed through language, perceptual similarity and perceptual interactions during information processing.” Consistent with this line of argument (that crossmodal correspondences can be considered as a “weak” form of synesthesia), Stevenson and Tomiczek (2007) argued that the phenomenon of certain food-related olfactory stimuli taking on taste properties as a ubiquitous form of olfactory-induced synesthesia (see also Stevenson & Boakes, 2004). Notice here how such an account, should it be accepted, does not necessitate that the synesthetically related olfactory and gustatory stimuli are perceptually similar. Note that in synesthesia proper, there is no sense in which the inducer and concurrent are thought of as being perceptually similar.

Nevertheless, Stevenson and Tomiczek’s (2007) suggestion has been robustly questioned by Auvray and Spence (2008) and Deroy and Spence (2013). Two key issues to bear in mind here are first that the connection between olfactory stimuli and gustatory properties is not idiosyncratic between individuals, and second that people tend to experience a unitary flavor rather than a distinction between inducer and concurrent that is such a signature feature of synesthesia proper (see Spence, 2015). For these and several other reasons, the description of OITE as a ubiquitous form of synesthesia would seem misleading and hence should probably be abandoned. Nevertheless, the point remains that while crossmodal correspondences might potentially be based on the perceptual similarity of the corresponding stimuli, they need not be (see Spence & Di Stefano, 2022).

**Figure 2.**
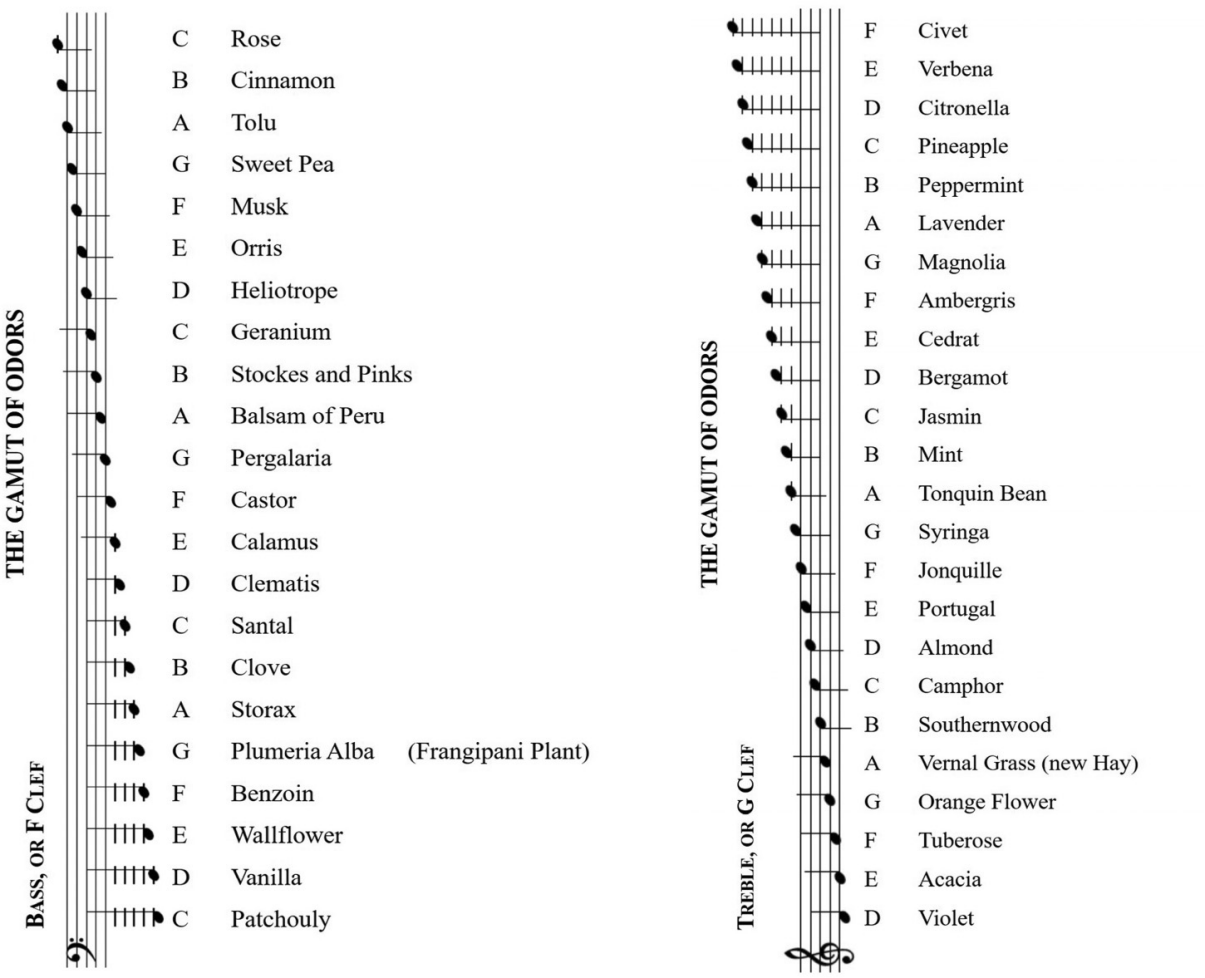
Scale of crossmodal correspondences between sound and odors reproduced from Piesse (1867, pp. 42-43).

Over the years, commentators have proposed a number of more or less surprising crossmodal correspondences between olfactory and both auditory and visual stimuli (see Deroy et al., 2013, for a review). So, for example, the 19th century chemist/perfumer Septimus Piesse (1867, 1891) once famously made a connection between a range of 24 scents and different musical notes in his so-called “Gamut of odours” (see Figure 2). Meanwhile, Von Hornbostel (1931) put forward the suggestion that sensory brightness should be considered as a universal (or amodal) dimension of sensory experience (see also Hartshorne, 1934).^6^ That said, a few years later, the North American psychologist Cohen (1934) argued that all that was needed was in fact ratio properties amongst analogs unisensory dimensions (i.e., the data did not necessarily support the existence of a universal, or amodal, dimension of sensory brightness; see also Ellermeier et al., 2021, on the ratio-based crossmodal matching of visual brightness and sound intensity; cf. Heller, 2021; Luce et al., 2010).^7^

### Aligning Perceptual Dimensions

**Figure 3.**
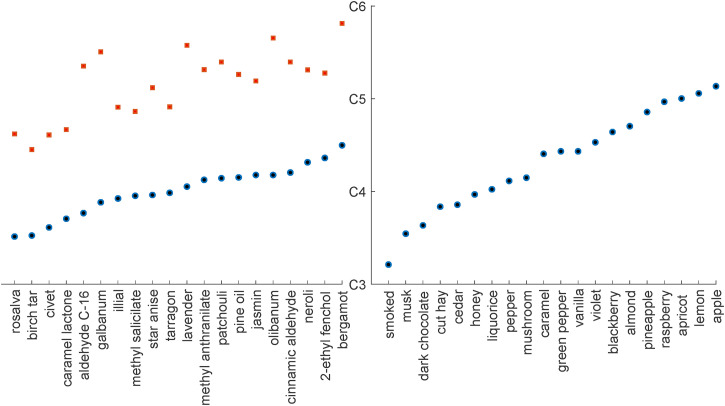
Crossmodal matchings (or correspondences) between odors and pitch. Adapted from Belkin et al. (1997) (left) and Crisinel and Spence (2012) (right).

Several researchers have attempted to document crossmodal correspondences between specific olfactory stimuli and the dimension of auditory pitch (Belkin et al., 1997; Crisinel & Spence, 2012), though intriguingly in Belkin et al.’s case, neither pleasantness, nor intensity, provided a satisfactory explanation of the data. Meanwhile Gilbert et al. (1996; see also Kemp & Gilbert, 1997) were able to demonstrate an inverse correlation between the dimensions of lightness and olfactory intensity (see Figure 3).^8^ More recently, Gilbert et al. (2016) suggested that color-olfactory crossmodal mappings could be explained on the basis of emotional mediation. Note, though, that in the latter case, the color stimuli that participants had to choose between varied in terms of a range of dimensions (i.e., hue, saturation, and lightness). Certainly, the notion of emotionally mediated crossmodal correspondences has become increasingly popular in recent years (see Cunningham & Weinel, 2016; Hauck et al., 2022; Spence, 2020a; Spence & Di Stefano, 2022).

## On the Similarities and Differences Between Orthonasal and Retronasal Olfaction

A number of researchers have wanted to distinguish between orthonasal and retronasal smell. Orthonasal sniffing occurs when we inhale odors from the external environment, while retronasal olfaction occurs when odors are pulsed out from the oral cavity to the back of the nose when chewing and swallowing (Fincks, 1886; Masaoka et al., 2010; Patrick, 1899; Rozin, 1982; Wilson, 2021). Rozin has written of the “duality” of the sense of smell. However, few other researchers have gone so far as to suggest that orthonasal and retronasal smell should be considered as distinct senses (or sensory systems). That said, a growing body of cognitive neuroscience research has, in recent years, demonstrated a somewhat distinct recruitment of neural areas in the case of the two senses of smell (e.g., Blankenship et al., 2019; Chapuis et al., 2009; Small et al., 2005; Veldhuizen et al., 2010; see also Gagnon et al., 2015). So, for example, Blankenship et al. have shown that retronasal olfaction requires taste cortex whereas orthonasal olfaction does not.

Small et al. (2005) conducted a neuroimaging study in which they compared the odor of chocolate (i.e., food-related) with three non-food odors. When the chocolate odor was compared to the other olfactants, orthonasal–retronasal stimulation revealed enhanced thalamic, insular, orbitofrontal cortex (OFC), hippocampal and amygdalar activity—all were more active when sniffing chocolate odour. The retronasal–orthonasal comparison, meanwhile, revealed more definitive activity in OFC, along with a number of brain areas putatively involved in food reward (temporal gyrus, temporal operculum, periegnual cingulate, etc.). Small and her colleagues summarized their findings in terms of the operation of “wanting” (orthonasal) and “liking” (retronasal)—“the amygdala representing anticipation of food reward and the temporal gyrus and others reflecting the receipt of food reward.”

Given the growing body of evidence suggesting that different neural circuits are involved in the orthonasal and retronasal experience of odors, and the suggestion that these might be considered as constituting two senses of smell, one might legitimately want to question the similarity between orthonasal smell, for example, of a strawberry and the retronasal smell, or better said, retronasal contribution to the flavor of strawberry (Spence et al., 2015). At the same time, however, it is striking how rarely we are aware of any perceptual difference between odors when experienced orthonasally versus retronasally. So similar are they, in fact, that people are rarely even aware that there might be a meaningful difference between them (Pierce & Halpern, 1996; Sun & Halpern, 2005; Voirol & Daget, 1986; though see Diaz, 2004, for occasional differences in perceived stimulus intensity between the orthonasal and retronasal routes). As Stevenson (2009, p. 231) puts it, while there are clear differences, the similarities “between orthonasal and retronasal perception are predominant.”

In fact, the exceptions, such as freshly ground coffee (which is often reported to smell orthonasally great, but be disappointing when experienced retronasally when drinking the coffee) and certain cheeses (such as Époisses; which may smell absolutely terrible, but deliver a highly desirable retronasal flavor) are noticeable by their rarity. To this list one might also want to add the so-called stinking tofu, and the legendary durian fruit of South-East Asia (see Stevenson et al., 2007, on the latter). In such cases, it is unclear whether the multiple associations (or referents that such odorants are linked to) is the key difference (such as isolaveric acid being a distinctive component of both certain cheeses, but also sweaty trainers, etc.), or whether instead certain volatiles may be “stripped” from the retronasal aroma by saliva (see Bonnans & Noble, 1995; Ge, 2012), meaning that the composition of the orthonasal and retronasal olfactory stimuli is actually physically different hence explaining the different affective associations (cf. Goldberg et al., 2018; Hannum, Stegman, Fryer, & Simons, 2018). Nevertheless, if one takes the suggestion that can be seen as emerging from Rozin’s (1982) paper that there are two senses of smell seriously then one might legitimately want to discuss the very high degree of perceptual similarity between the orthonasal and retronasal experience of the vast majority of odors. However, it is worth noting that in the four decades since Rozin published his paper, I am not aware of any other researcher who have wished to pursue the line that there are two distinct senses of smell (as might be needed to talk about the perceptual similarity of the orthonasal and retronasal senses of smell in a similar way that we have been discussing the perceptual similarity of olfactory and gustatory stimuli.

## Featural Versus Dimensional Accounts of Similarity

The question of whether a dimensional or feature-based account of similarity is more appropriate in the case of the chemical senses is by no means obvious. In his classic article, Tversky (1977, p. 328) notes that: “It has been argued by many authors that dimensional representations are appropriate for certain stimuli (e.g., colors, tones) but not for others. It seems more appropriate to represent faces, countries, or personalities in terms of many qualitative features than in terms of a few quantitative dimensions. The assessment of similarity between such stimuli, therefore, may be better described as a comparison of features rather than as the computation of metric distance between points.”

One theoretical issue that challenges any idea of perceptual similarity that might be based on shared phenomenological properties relates to the fact that a thing might be an “A-thing” with respect to “A-ness,” and at the same time might be a “B-thing” with respect to “B-ness” (see Rodriguez-Pereyra, 2002). Such critics point to the fact that any two objects might share at least one phenomenological property and thus, as Goodman (1972) has argued, similarity would simply be a universal relation—namely, everything would be similar to everything else—and therefore claims regarding similarity would become somehow meaningless. Moreover, different properties count differently as far as perceptual similarity is concerned. For example, a tomato is more similar to grapes than to blood, despite both tomatoes and blood being red. Thus, perceptual similarity would appear to depend on more than just a simple count of shared and unshared perceptual features or attributes. One might also struggle to define featural properties of the basic tastes.

Meanwhile, according to Licon et al. (2018), pleasantness and the presence of trigeminal sensations, and particularly irritation, appear to act as salient dimensions in organizing the semantic and physiological spaces of odors thus potentially supporting a dimensional account of olfactory similarity. However, given the continuing disagreement concerning the existence of fundamental dimensions, or categories, of olfactory experience, and whether the space can be captured by only a few dimension or reflects a multi-dimensional space (e.g., see Berglund et al., 1973; Castro et al., 2013; Koulakov et al., 2011; Meister, 2015; Schiffman, 1974; Schiffman et al., 1977; Zarzo, 2008), one might assume that a dimensional representation of olfactory similarity would not necessarily provide the most appropriate way in which to conceptualize olfactory similarity.^9^ As such, the possibility should perhaps be considered that a feature-based matching account might turn out to provide a more parsimonious explanation of similarity data as far as the chemical senses are concerned (or at the very least in the cases of olfaction and flavor; though see Jones et al., 1978, for uncertainty over the appropriateness of such an approach).^10^

## Should “Sensory Intricacy” be Considered as Another Kind of Similarity

One might also consider whether complexity, or the more recently introduced notion of sensory intricacy might also provide an alternative means of matching stimuli across the senses. In this regard, Snitz et al. (2016) conducted an intriguing study of sensory intricacy involving people assessing both olfactory and visual stimuli. In this case, “sensory intricacy” was measured in terms of the degree of disagreement between participants using a version of the Semantic Differential Technique (SDT; Osgood et al., 1957; Snider & Osgood, 1969). Here, though, it is worth noting that the latter approach delivers a judgment that is based on affective/connotative (rather than perceptual) meaning. What is more, judging stimuli presented in different sensory modalities as similar because they share a similar degree of intricacy (note here that Snitz and colleagues argue that “sensory intricacy” is distinct from stimulus complexity; see Spence & Wang, 2018) would be neither a perceptually, nor an affectively-based judgment. At the same time, however, it is unclear whether people necessarily have any kind of subjective awareness of the sensory intricacy of a given stimulus (i.e., this characterization may only emerge when data are analyzed at the group level). Nevertheless, this clearly leaves open the possibility of perceived complexity of the component stimuli as providing another grounds for combining, or rather pairing, stimuli across the chemical senses (see Spence, 2020b).

## Conclusions

Given the evidence that has been reviewed here, it is tempting to suggest that Helmholtz (1878/1971) may have been right after all; His claim that it is simply not possible to make judgments of (perceptual) similarity would appear to be broadly true, with the notable exception of food-related odors and tastes (i.e., perceptual similarity judgments within the chemical senses; and/or between orthonasal and retronasal smell should they be considered as distinct senses; see Hannum et al., 2018; Rozin, 1982). That said, other researchers have expressed no hesitation about asserting the possibility of crossmodal judgments of perceptual similarity (e.g., Marks, 2011).^11^ Certainly, the majority of crossmodal correspondences would appear to be based on association (i.e., the internalization of the statistical regularities of the environment) rather than necessarily on perceptual similarity. Emotional mediation, or common affective/connotative meaning as revealed by, for example, research involving the Semantic Differential technique (see Dalton et al., 2008), often appears to play key role in determining people’s judgments of the similarity of stimuli from different categories (e.g., Gilbert et al., 2016; Pedović & Stosić, 2018). Perhaps, though, we need to accept the possibility that crossmodal correspondences between (at least food-related) olfactory stimuli and basic tastes (i.e., gustatory qualities), are somehow importantly different from the correspondence that many people experience between other pairings of modalities (i.e., such as between visual and auditory stimuli, see Spence, 2022b). Different in the sense that judgments of perceptual similarity between certain pairs of olfactory and gustatory stimuli might make sense in a way that they do not when it comes to any other pairing of sensory modalities.

At the same time, however, there is no need to go so far as to suggest that the close connection between food-related odors and the dominant tastes that they so often co-occur with in foods should be considered as a ubiquitous form of olfactory-gustatory synesthesia, as suggested by Stevenson and Tomiczek (2007; and see Auvray & Spence, 2008; Deroy & Spence, 2013, for arguments against accepting this suggestion). Ultimately, therefore, the fact that food-related olfactory stimuli can sometimes take on the perceptual qualities of the basic taste with which they are so often associated in foods (such as vanilla becoming (associated with) sweetness, despite the fermented vanilla bean itself tasting bitter; see Spence, submitted) means that crossmodal judgments of perceptual similarity may be possible in the case of certain olfactory–gustatory combinations in a way that is simply not possible for any other combination of sensory inputs. That being said it should also be remembered that affective, or emotionally-mediated, similarity likely operates across any pairing of sensory dimensions, and often represents the most plausible basis for establishing a connection, or correspondence, between unrelated sensations.

## Notes

1. Cited more than 10,000 times last time I checked.

2. This term loosely defined (see Spence, 2021, on this point).

3. Though, in truth, this would presumably be difficult for the participants to ascertain subjectively (see Rozin, 1982; Spence, 2016; Spence et al., 2015).

4. Schifferstein and Verlegh (1996, p. 90) define congruency as “the extent to which two stimuli are appropriate for combination in a food product.” Seemingly hinting at a similar dissociation between similarity and congruency, Blank and Mattes (1990, p. 292) note that: “The results indicate that spices do not necessarily have to taste sweet to be perceived as having properties compatible with sweetness. That is, whereas some spices may not seem to have an overt sweet taste, their use in sweet foods and beverages may make them more acceptable in terms of a pleasing flavour, e.g., adding nutmeg to custard.”

5. Perceptual similarity in the chemical senses is likely also culturally determined (see Spence, 2022a, submitted, on this point).

6. The amodal basis of at least certain crossmodal correspondences also appears in the work of Eleanor Gibson (1969). She writes: “Information for [amodal features] may be extracted from more than one kind of sensory experience. Perhaps all cross-modal similarity is amodal in that sense. We need, however, to remind ourselves that these similarities do not necessarily require an explanation based on association.” continuing: “That there are relationships between the sensory system is obvious: Intersensory similarity is responsible for much of poetic metaphor. Emily Dickinson, for examples, invokes similarities between three modes of experience in the following stanza: There’s a certain slant of light; on winter afternoons; that oppresses, like the weight; of cathedral tunes (Gibson, 1969, p. 215). Later, she writes that: “Similarities of qualities of different modes has already been mentioned. We need, however, to remind ourselves that these similarities do not necessarily require an explanation based on association. How would association explain the similarity of an oppressive weight and an oppressive cathedral tune?” (Gibson, 1969, p. 219).

7. Relevant here, it is worth noting how individual differences in taster status might be expected to modulate the crossmodal matching of taste to sound intensity (Marks et al., 1988).

8. Though see Marks (1974) on the bimodal nature of loudness-brightness correspondences within the population.

9. See Schiffman and Erickson (1971, 1980) for a discussion of the most appropriate psychophysical model to describe gustatory quality (see also Gregson, 1965; Schiffman & Falkenberg, 1968; Yoshida, 1963).

10. Consistent with such a feature-based account of similarity, Ravia et al. (2020) created a number of olfactory metamers. According to these researchers, the perceived similarity between odours could be predicted using a model with 21 physicochemical features.

11. That said, Marks makes no specific claims regarding the pairing of olfactory and gustatory stimuli.

## References

[bibr1-20416695221124154] Amsellem S. Ohla K. (2016). Perceived odor-taste congruence influences intensity and pleasantness differently. Chemical Senses, 41(8), 677–684. 10.1093/chemse/bjw078 27384192

[bibr2-20416695221124154] Aristotle (1907). De Anima, translated by R. D. Hicks. Cambridge University Press.

[bibr3-20416695221124154] Auvray M. Spence C. (2008). The multisensory perception of flavor. Consciousness and Cognition, 17(3), 1016–1031. 10.1016/j.concog.2007.06.005 17689100

[bibr4-20416695221124154] Baeyens F. Eelen P. Van den Bergh O. Crombez G. (1990). Flavor-flavor and color-flavor conditioning in humans. Learning and Motivation, 21(4), 434–455. 10.1016/0023-9690(90)90025-J

[bibr5-20416695221124154] Barlow H. (2001). The exploitation of regularities in the environment by the brain. Behavioral and Brain Sciences, 24(4), 602–607. 10.1017/S0140525X01000024. 12048943

[bibr6-20416695221124154] Belkin K. Martin R. Kemp S. E. Gilbert A. N. (1997). Auditory pitch as a perceptual analogue to odor quality. Psychological Science, 8(4), 340–342. 10.1111/j.1467-9280.1997.tb00450.x

[bibr7-20416695221124154] Berglund B. Berglund D. Engen T. Ekman G. (1973). Multidimensional analysis of twenty-one odors. Scandinavian Journal of Psychology, 14(1), 131–137. 10.1111/j.1467-9450.1973.tb00104.x. 4705857

[bibr8-20416695221124154] Blank D. M. Mattes R. D. (1990). Sugar and spice: Similarities and sensory attributes. Nursing Research, 39(5), 290–293. PMID: 2399134. 2399134

[bibr9-20416695221124154] Blankenship M. L. Grigorova M. Katz D. B. Maier J. X. (2019). Retronasal odor perception requires taste cortex, but orthonasal does not. Current Biology, 29(1), 62–69.e3. 10.1016/j.cub.2018.11.011. 30581018 PMC6604050

[bibr10-20416695221124154] Bonnans S. R. Noble A. C. (1995). Interaction of salivary flow with temporal perception of sweetness, sourness, and fruitiness. Physiology & Behavior, 57(3), 569–574. 10.1016/0031-9384(94)00367-E 7753896

[bibr11-20416695221124154] Castro J. B. Ramanathan A. Chennubhotla C. S. (2013). Categorical dimensions of human odor descriptor space revealed by non-negative matrix factorization. PLoS One, 8(9), e73289. 10.1371/journal.pone.0073289. 24058466 PMC3776812

[bibr12-20416695221124154] Chapuis J. Garcia S. Messaoudi B. Thevenet M. Ferreira G. Gervais R. Ravel N. (2009). The way an odor is experienced during aversive conditioning determines the extent of the network recruited during retrieval: A multisite electrophysiological study in rats. The Journal of Neuroscience, 29(33), 10287–10298. 10.1523/JNEUROSCI.0505-09.2009. 19692603 PMC6665786

[bibr13-20416695221124154] Cleland T. A. Morse A. Yue E. L. Linster C. (2002). Behavioral models of odor similarity. Behavioral Neuroscience, 116(2), 222–231. 10.1037/0735-7044.116.2.222. 11996308

[bibr14-20416695221124154] Cohen N. E. (1934). Equivalence of brightness across modalities. American Journal of Psychology, 46, 117–119.

[bibr15-20416695221124154] Coucquyt P. Lahousse B. Langenbick J. (2020). The art and science of Foodpairing: 10,000 flavour matches that will transform the way you eat. Mitchell Beazley.

[bibr16-20416695221124154] Crisinel A.-S. Spence C. (2012). A fruity note: Crossmodal associations between odors and musical notes. Chemical Senses, 37(2), 151–158. 10.1093/chemse/bjr085. 21852708

[bibr17-20416695221124154] Cunningham S. Weinel J. (2016). The sound of the smell (and taste) of my shoes too: Mapping the senses using emotion as a medium. In *AM ‘16: Proceedings of the Audio Mostly* 2016, October 2016 (pp. 28–33). ACM Press. 10.1145/2986416.2986456.

[bibr18-20416695221124154] Dalton P. Maute C. Oshida A. Hikichi S. Izumi Y. (2008). The use of semantic differential scaling to define the multi-dimensional representation of odors. Journal of Sensory Studies, 23(4), 485–497. 10.1111/j.1745-459X.2008.00167.x. 19122880 PMC2533099

[bibr19-20416695221124154] Deroy O. Crisinel A.-S. Spence C. (2013). Crossmodal correspondences between odors and contingent features: Odors, musical notes, and geometrical shapes. Psychonomic Bulletin & Review, 20, 878–896. 10.3758/s13423-013-0397-0. 23463615

[bibr20-20416695221124154] Deroy O. Spence C. (2013). Why we are not all synesthetes (not even weakly so). Psychonomic Bulletin & Review, 20, 643–664. 10.3758/s13423-013-0387-2. 23413012

[bibr21-20416695221124154] Diaz M. E. (2004). Comparison between orthonasal and retronasal flavour perception at different concentrations. Flavour and Fragrance Journal, 19(6), 499–504.

[bibr22-20416695221124154] Ellermeier W. Kattner F. Raum A. (2021). Cross-modal commutativity of magnitude productions of loudness and brightness. Attention, Perception, & Psychophysics, 83(7), 2955–2967. 10.3758/s13414-021-02324-y. PMC8460511 34105093

[bibr23-20416695221124154] Everson S. (1995). Proper sensibles and Καθ᾽ Αὑτά causes. Phronesis, 40(3), 265–292. http://www.jstor.org/stable/4182505.

[bibr24-20416695221124154] Fernandez M. Bahrick L. E. (1994). Infants’ sensitivity to arbitrary object-odor pairings. Infant Behavior and Development, 17(4), 471–474. 10.1016/0163-6383(94)90040-X

[bibr25-20416695221124154] Fial A. Z. (1978). Adding spice to sugar-reduced diets. Journal of the American Dietetic Association, 73, 658–659. 722016

[bibr26-20416695221124154] Fincks H. T. (1886). The gastronomic value of odours. Contemporary Review, 50, 680–695.

[bibr27-20416695221124154] Foroni F. Pergola G. Rumiati R. I. (2016). Food color is in the eye of the beholder: The role of human trichromatic vision in food evaluation. Scientific Reports, 6, 37034. 10.1038/srep37034. 27841327 PMC5107980

[bibr28-20416695221124154] Frank R. A. Shaffer G. Smith D. V. (1991). Taste-odor similarities predict taste enhancement and suppression in taste-odor mixtures. Chemical Senses, 16, 523.

[bibr29-20416695221124154] Gagnon L. Ismaili A. R. A. Ptito M. Kupers R. (2015). Superior orthonasal but not retronasal olfactory skills in congenital blindness. PLoS One, 10, e0122567. 10.1371/journal.pone.0122567 25822780 PMC4379017

[bibr30-20416695221124154] Ge L. (2012). Why coffee can be bittersweet. *FT Weekend Magazine*, October 13/14th, 50.

[bibr31-20416695221124154] Gibson E. J. (1969). Principles of perceptual learning and development. Appleton.

[bibr32-20416695221124154] Gilbert A. N. Fridlund A. J. Lucchina L. A. (2016). The color of emotion: A metric for implicit color associations. Food Quality & Preference, 52, 203–210. 10.1016/j.foodqual.2016.04.007

[bibr33-20416695221124154] Gilbert A. N. Martin R. Kemp S. E. (1996). Cross-modal correspondence between vision and olfaction: The color of smells. American Journal of Psychology, 109(3), 335–351. 10.2307/1423010 8837406

[bibr34-20416695221124154] Goldberg E. M. Wang K. Goldberg J. Aliani M. (2018). Factors affecting the ortho- and retronasal perception of flavors: A review. Critical Reviews in Food Science and Nutrition, 58(6), 913–923. 10.1080/10408398.2016.1231167. 27646486

[bibr35-20416695221124154] Goodman N. (1972). Seven strictures on similarity. In Problems and projects (pp. 437–446). Bobs-Merrill.

[bibr36-20416695221124154] Gregson R. A. M. (1965). Representation of taste mixture cross-modal matching in a Minkowski r-metric. Australian Journal of Psychology, 17(3), 195–204. 10.1080/00049536508255196.

[bibr110-20416695221124154] Hannum, M., Stegman, M. A., Fryer, J. A., & Simons, C. T. (2018). Different olfactory percepts evoked by orthonasal and retronasal odorant delivery, *Chemical Senses*, *43*(7), 515–521. 10.1093/chemse/bjy043 29982522

[bibr37-20416695221124154] Hartshorne C. (1934). The philosophy and psychology of sensation. University of Chicago Press.

[bibr38-20416695221124154] Hauck P. von Castell C. Hecht H. (2022). Crossmodal correspondence between music and ambient color is mediated by emotion. Multisensory Research, 10.1163/22134808-bja10077. 35985652

[bibr39-20416695221124154] Heller J. (2021). Internal references in cross-modal judgments: A global psychophysical perspective. Psychological Review, 128(3), 509–524. 10.1037/rev0000280. 33939457

[bibr40-20416695221124154] Helmholtz H. v. (1878/1971). Treatise on physiological optics (vol. II). Dover Publications.

[bibr41-20416695221124154] Huisman G. Bruijnes M. Heylen D. K. J. (2016). A moving feast: Effects of color, shape and animation on taste associations and taste perceptions. *MHFI ‘16 Proceedings of the 1st Workshop on Multi-sensorial Approaches to Human-Food Interaction*. Article No. 4 Tokyo, Japan. November 16, 2016. New York, NY: ACM.

[bibr42-20416695221124154] Jones F. N. Roberts K. Holman E. W. (1978). Similarity judgments and recognition memory for some common spices. Perception & Psychophysics, 24, 2–6. 10.3758/BF03202967 693237

[bibr43-20416695221124154] Kemp S. E. Gilbert A. N. (1997). Odor intensity and color lightness are correlated sensory dimensions. American Journal of Psychology, 110(1), 35–46. 9100340

[bibr44-20416695221124154] Koulakov A. A. Kolterman B. E. Enikolopov A. G. Rinberg D. (2011). In search of the structure of human olfactory space. Frontiers in Systems Neuroscience, 5, 65. 10.3389/fnsys.2011.00065. 21954378 PMC3173711

[bibr45-20416695221124154] Licon C. C. Manesse C. Dantec M. Fournel A. Bensafi M. (2018). Pleasantness and trigeminal sensations as salient dimensions in organizing the semantic and physiological spaces of odors. Scientific Reports, 8, 8444. 10.1038/s41598-018-26510-5. 29855500 PMC5981304

[bibr46-20416695221124154] Luce R. D. Steingrimsson R. Narens L. (2010). Are psychophysical scales of intensities the same or different when stimuli vary on other dimensions? Theory with experiments varying loudness and pitch. Psychological Review, 117, 1247–1258. 10.1037/a0020174. 20836612

[bibr47-20416695221124154] Marks L. E. (1974). On associations of light and sound: The mediation of brightness, pitch, and loudness. The American Journal of Psychology, 87(1/2), 173–188. 10.2307/1422011 4451203

[bibr48-20416695221124154] Marks L. E. (2011). Synesthesia, then and now. Intellectica, 55, 47–80. 10.3406/intel.2011.1161

[bibr49-20416695221124154] Marks L. E. Stevens J. C. Bartoshuk L. M. Gent J. F. Rifkin B. Stone V. K. (1988). Magnitude-matching: The measurement of taste and smell. Chemical Senses, 13(1), 63–87. 10.1093/chemse/13.1.63

[bibr50-20416695221124154] Martino G. Marks L. E. (2001). Synesthesia: Strong and weak. Current Directions in Psychological Science, 10(2), 61–65. 10.1111/1467-8721.00116

[bibr51-20416695221124154] Masaoka Y. Satoh H. Akai L. Homma I. (2010). Expiration: The moment we experience retronasal olfaction in flavor. Neuroscience Letters, 473(2), 92–96. 10.1016/j.neulet.2010.02.024. 20171264

[bibr52-20416695221124154] Mehrabi A. Dixon S. Sandler M. (2019). Vocal imitation of percussion sounds: On the perceptual similarity between imitations and imitated sounds. PLoS ONE, 14(7), e0219955. 10.1371/journal.pone.0219955. 31344080 PMC6657857

[bibr53-20416695221124154] Meister M. (2015). On the dimensionality of odor space. eLife, 4, e07865. 10.7554/eLife.07865 26151672 PMC4491593

[bibr54-20416695221124154] Murphy C. Cain W. S. (1980). Taste and olfaction: Independence vs. interaction. Physiology and Behavior, 24, 601–605. 10.1016/0031-9384(80)90257-7 7375580

[bibr55-20416695221124154] O’Mahony M. (1983). Gustatory responses to nongustatory stimuli. Perception, 12(5), 627–633. 10.1068/p120627 6676714

[bibr56-20416695221124154] Osgood C. E. Suci G. J. Tannenbaum P. H. (1957). The measurement of meaning. University of Illinois Press.

[bibr111-20416695221124154] Owens, J. (1982). Aristotle on common sensibles and incidental perception. *Phoenix*, *36*(3), 215–236. 10.2307/1087890

[bibr57-20416695221124154] Patrick G. T. W. (1899). On the confusion of tastes and odors (from the proceedings of the seventh annual meeting of the American Psychological Association, Columbia University, New York, December, 1898). Psychological Review, 6, 160–162.

[bibr58-20416695221124154] Pedović I. Stosić M. (2018). Předběžná sdělení: A comparison of verbal and sensory presentation methods in measuring crossmodal correspondence within a semantic-based approach. Československá Psychologie, LXII(6), 602–615.

[bibr59-20416695221124154] Pierce J. Halpern B. P. (1996). Orthonasal and retronasal odorant identification based upon vapor phase input from common substances. Chemical Senses, 21(5), 529–543. 10.1093/chemse/21.5.529 8902282

[bibr60-20416695221124154] Piesse G. W. S. (1867). The art of perfumery and the methods of obtaining the odors of plants: With instructions for the manufacture of perfumes for the handkerchief, scented powders, odorous vinegars, dentifrices, pomatums, cosmetics, perfumed soap, etc., to which is added an appendix on preparing artificial fruit-essences, etc. Lindsay & Blakiston.

[bibr61-20416695221124154] Piesse G. W. S. (1891). Piesse’s art of perfumery (5th ed.). Piesse and Lubin. Downloaded from http://www.gutenberg.org/files/16378/16378-h/16378-h.htm.

[bibr62-20416695221124154] Ravia A. Snitz K. Honigstein D. Finkel M. Zirler R. Perl O. Secundo L. Laudamiel C. Harel D. Sobel N. (2020). A measure of smell enables the creation of olfactory metamers. Nature, 588, 118–123. 10.1038/s41586-020-2891-7. 33177711

[bibr63-20416695221124154] Reardon P. Bushnell E. W. (1988). Infants’ sensitivity to arbitrary pairings of color and taste. Infant Behavior and Development, 11(2), 245–250. 10.1016/S0163-6383(88)80010-9

[bibr64-20416695221124154] Rodriguez-Pereyra G. (2002). Resemblance nominalism: A solution to the problem of universals. Oxford University Press.

[bibr65-20416695221124154] Rozin P. (1982). “Taste-smell confusions” and the duality of the olfactory sense. Perception & Psychophysics, 31, 397–401. 10.3758/BF03202667 7110896

[bibr66-20416695221124154] Schifferstein H. N. J. Verlegh P. W. J. (1996). The role of congruency and pleasantness in odor-induced taste enhancement. Acta Psychologica, 94(1), 87–105. 10.1016/0001-6918(95)00040-2 8885712

[bibr67-20416695221124154] Schiffman S. S. (1974). Physiochemical correlates of olfactory quality. Science, 185(4146), 112–117. 10.1126/science.185.4146.112 4834219

[bibr68-20416695221124154] Schiffman H. Falkenberg P. (1968). The organization of stimuli and sensory neurons. Physiology & Behavior, 3, 197–201. 10.1016/0031-9384(68)90054-1.

[bibr69-20416695221124154] Schiffman S. S. Erickson R. P. (1971). A psychophysical model of gustatory quality. Physiology & Behavior, 7(4), 617–633. 10.1016/0031-9384(71)90117-x 5131220

[bibr70-20416695221124154] Schiffman S. S. Erickson R. P. (1980). The issue of primary tastes versus a taste continuum. Neuroscience and Biobehavioral Reviews, 4(2), 109–117. 10.1016/0149-7634(80)90009-3 7422163

[bibr71-20416695221124154] Schiffman S. S. Robinson D. E. Erickson R. P. (1977). Multidimensional scaling of odorants: Examination of psychological and physicochemical dimensions. Chemical Senses and Flavor, 2(3), 375–390. 10.1093/chemse/2.3.375

[bibr72-20416695221124154] Small D. M. Gerber J. C. Mak Y. E. Hummel T. (2005). Differential neural responses evoked by orthonasal versus retronasal odorant perception in humans. Neuron, 47(4), 593–605. 10.1016/j.neuron.2005.07.022. 16102541

[bibr73-20416695221124154] Snider J. G. Osgood C. E. (1969). Semantic differential technique: A sourcebook. Aldine Publishing.

[bibr74-20416695221124154] Snitz K. Arzi A. Jacobson M. Secundo L. Weissler K. Yablonka A. (2016). A cross modal performance-based measure of sensory stimuli intricacy. PLoS ONE, 11(2), e0147449. 10.1371/journal.pone.0147449. 26840072 PMC4740424

[bibr75-20416695221124154] Spence C. (2011). Crossmodal correspondences: A tutorial review. Attention, Perception, & Psychophysics, 73, 971–995. 10.3758/s13414-010-0073-7. 21264748

[bibr76-20416695221124154] Spence C. (2015). Just how much of what we taste derives from the sense of smell? Flavour, 4, 30. 10.1186/s13411-015-0040-2.

[bibr77-20416695221124154] Spence C. (2016). Oral referral: On the mislocalization of odours to the mouth. Food Quality & Preference, 50, 117–128. 10.1016/j.foodqual.2016.02.006.

[bibr78-20416695221124154] Spence C. (2020a). Assessing the role of emotional mediation in explaining crossmodal correspondences involving musical stimuli. Multisensory Research, 33, 1–29. 10.1163/22134808-20191469. 31648195

[bibr79-20416695221124154] Spence C. (2020b). Flavour pairing: A critical review of the literature on food and beverage pairing. Food Research International, 133, 109124. 10.1016/j.foodres.2020.109124. 32466920

[bibr80-20416695221124154] Spence C. (2020c). Olfactory-colour crossmodal correspondences in art, science, & design. Cognitive Research: Principles & Implications (CRPI), 5, 52. 10.1186/s41235-020-00246-1. https://rdcu.be/b9oDJ. 33113051 PMC7593372

[bibr81-20416695221124154] Spence C. (2021). Gastrophysics: The psychology of herbs and spices. In McWilliams M. (Ed.), Proceedings of the Oxford symposium on food and cookery, 2020 (pp. 11–40). Prospect Books.

[bibr82-20416695221124154] Spence C. (2022a). Factors affecting odour-induced taste enhancement. Food Quality & Preference, 96, 104393. 10.1016/j.foodqual.2021.104393.

[bibr83-20416695221124154] Spence C. (2022b). Gastrophysics: Getting creative with pairing flavours. International Journal of Gastronomy & Food Science, 27, 100433. 10.1016/j.ijgfs.2021.100433.

[bibr84-20416695221124154] Spence C. (submitted). Why should vanilla be the most liked smell? Nature Food.

[bibr85-20416695221124154] Spence C. Di Stefano N. (2022). Coloured hearing, colour music, colour organs, and the search for perceptually meaningful correspondences between colour and pitch. i-Perception, 13(3), 1–42. 10.1177/20416695221092802. PMC9099070 35572076

[bibr86-20416695221124154] Spence C. Di Stefano N. (submitted). What, if anything, can be considered amodal? Psychonomic Bulletin & Review. 10.3758/s13423-023-02447-3 PMC11543734 38381301

[bibr112-20416695221124154] Spence, C., & Levitan, C. A. (2022). Exploring the links between colours and tastes/flavours. *Journal of Perceptual Imaging (JPI)*, *4*(3):000408. 10.2352/J.Percept.Imaging.2022.5.000408

[bibr87-20416695221124154] Spence C. Wan X. Woods A. Velasco C. Deng J. Youssef J. Deroy O. (2015). On tasty colours and colourful tastes? Assessing, explaining, and utilizing crossmodal correspondences between colours and basic tastes. Flavour, 4, 23. 10.1186/s13411-015-0033-1.

[bibr88-20416695221124154] Spence C. Wang Q. J. (2018). On the meaning(s) of complexity in the chemical senses. Chemical Senses, 43(7), 451–461. 10.1093/chemse/bjy047 30010729

[bibr89-20416695221124154] Stevenson R. J. (2009). The psychology of flavour. Oxford University Press.

[bibr90-20416695221124154] Stevenson R. J. Boakes R. A. (2004). Sweet and sour smells: Learned synaesthesia between the senses of taste and smell. In Calvert G. A. Spence C. Stein B. E. (Eds.), The handbook of multisensory processing (pp. 69–83). MIT Press.

[bibr91-20416695221124154] Stevenson R. J. Tomiczek C. (2007). Olfactory-induced synesthesias: A review and model. Psychological Bulletin, 133(2), 294–309. 10.1037/0033-2909.133.2.294 17338601

[bibr92-20416695221124154] Stevenson R. J. Tomiczek C. Oaten M. (2007). Olfactory hedonic context affects both self-report and behavioural indices of palatability. Perception, 36(11), 1698–1708. 10.1068/p5726 18265849

[bibr93-20416695221124154] Sun B. C. Halpern B. P. (2005). Identification of air phase retronasal and orthonasal odorant pairs. Chemical Senses, 30(8), 693–706. 10.1093/chemse/bji062 16177226

[bibr94-20416695221124154] Tversky A. (1977). Features of similarity. Psychological Review, 84(4), 327–352. 10.1037/0033-295X.84.4.327

[bibr95-20416695221124154] Veldhuizen M. G. Nachtigal D. Teulings L. Gitelman D. R. Small D. M. (2010). The insular taste cortex contributes to odor quality coding. Frontiers in Human Neuroscience, 4, 58. 10.3389/fnhum.2010.00058. 20700500 PMC2917218

[bibr96-20416695221124154] Voirol E. Daget N. (1986). Comparative study of nasal and retronasal olfactory perception. Lebensmittel-Wissenschaft und Technologie, 19, 316–319.

[bibr97-20416695221124154] von Hornbostel E. M. (1931). Über Geruchshelligkeit [on smell brightness]. Pflügers Archiv für die Gesamte Physiologie des Menschen und der Tiere, 227, 517–538. 10.1007/BF01755351.

[bibr98-20416695221124154] Wegner-Clemens K. Malcolm G. L. Shomstein S. (2022). How much is a cow like a meow? A novel database of human judgements of audiovisual semantic relatedness. Attention, Perception, & Psychophysics, 84, 1317–1327. 10.3758/s13414-022-02488-1. 35449432

[bibr99-20416695221124154] Wilson K. A. (2021). Individuating the senses of ‘smell’: Orthonasal versus retronasal olfaction. Synthese, 2021, 10.1007/s11229-020-02976-7.

[bibr100-20416695221124154] Woods A. T. Spence C. (2016). Using single colours and colour pairs to communicate basic tastes. i-Perception, 7, 4. 10.1177/2041669516658817. PMC5030750 27698979

[bibr101-20416695221124154] Yearsley J. M. Pothos E. M. Barque-Duran A. Trueblood J. S. Hampton J. A. (2021). Context effects in similarity judgments. Journal of Experimental Psychology: General, 151(3), 711–717. 10.1037/xge0001097. 34472962

[bibr102-20416695221124154] Yoshida M. (1963). Similarity among different kinds of taste near the threshold concentration. Japanese Journal of Psychology, 34, 25–35. 10.4992/JJPSY.34.25

[bibr103-20416695221124154] Zarzo M. (2008). Psychologic dimensions in the perception of everyday odors: Pleasantness and edibility. Journal of Sensory Studies, 23(3), 354–376. 10.1111/j.1745-459X.2008.00160.x

